# Supplementary material for: Characterization of IgG Antibody Response against SARS-CoV-2 (COVID-19) in the Cypriot Population
Source: Microorganisms. 2021 Dec 31;10(1):85. doi: 10.3390/microorganisms10010085 (PMC8777616; doi:10.3390/microorganisms10010085)
Supplement: Supplementary file 1 [file microorganisms-10-00085-s001.zip › microorganisms-1437335-supplementary.pdf]

microorganisms

Supplementary materials

SUMMARY OF ASSAY PROCEDURE

Add 100 µl of sample to each well.

↓

Incubate at room temperature for 1 hour.

↓

Aspirate and wash each well three times.

↓

Add 100 µl of 1×Detection antibody solution to each well.

↓

Incubate at room temperature for 1 hour.

↓

Aspirate and wash each well four times.

↓

Add 100 µl of Substrate solution to each well.

↓

Incubate at room temperature for 15 minutes.

↓

Add 100 µl of Stop solution to each well.

↓

Measure absorbance of each well at 450 nm.

↓

Calculation and Interpretation

Figure S1. Summary of the ELISA protocol followed as described by the manufacturer. Kits used are the humanized IgG monoclonal antibody against SARS-CoV-2 Nucleocapsid Protein ELISA kit (SKU 41A227, Immunodiagnostics Ltd., Hong Kong) and humanized anti-S1RBD IgG monoclonal antibody ELISA kit (SKU 41A236, Immunodiagnostics Ltd., Hong Kong).

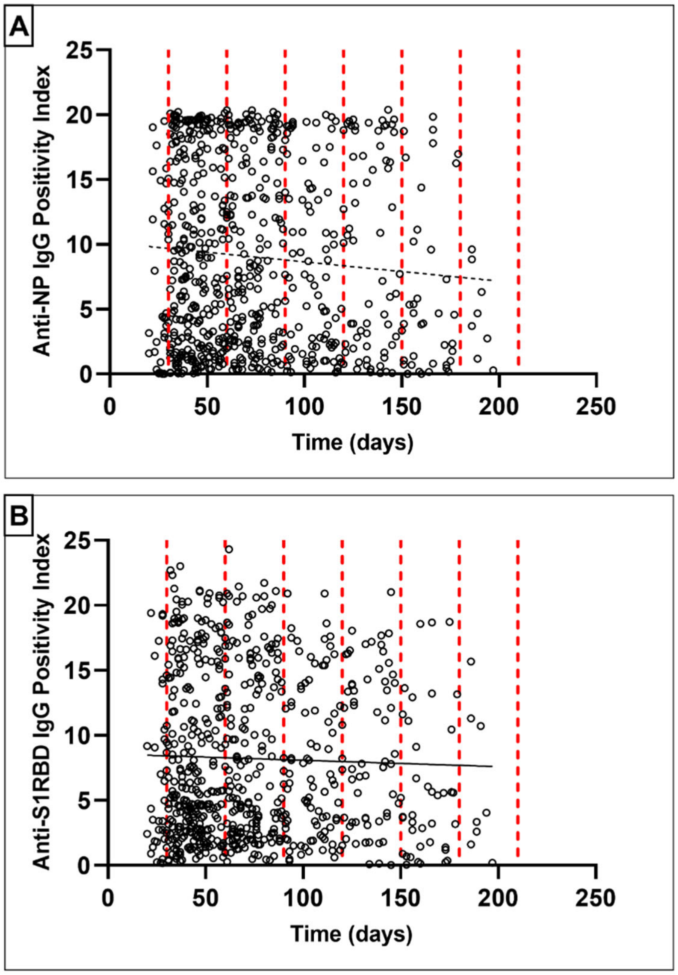

Figure S2. Change in antibody level across time. (**A**) Distribution of anti-NP IgG positivity index against time calculated (in days) as the time elapsed between the date of infection and the date of blood sampling (**B**) Distribution of anti-S1RBD IgG positivity index against time calculated (in days) as the time elapsed between the date of infection and the date of blood sampling. The space between vertical red dotted line represent 1-month intervals. A best fit line is constructed based on a linear regression model (dotted line in (**A**), and solid line in (**B**)). The decrease in (**A**) is shown to be significant ($p = 0.02$) while the decrease in (**b**) is not significant ($p = 0.40$).
